# Salivary Cortisol and Anxiety in Canadian Dentists over 1 Year of COVID-19

**DOI:** 10.1177/00220345231178726

**Published:** 2023-06-15

**Authors:** R.J. Kolbe, S.A. Madathil, L.M. Marin, R. Seth, N. Faraj, P.J. Allison, C. Quiñonez, M. Glogauer, W.L. Siqueira, M.F. Siqueira

**Affiliations:** 1 College of Dentistry, University of Saskatchewan, Saskatoon, Canada; 2 Faculty of Dental Medicine and Oral Health Sciences, McGill University, Montréal, Canada; 3 Faculty of Dentistry, University of Toronto, Toronto, Canada

**Keywords:** saliva, psychological distress, biomarkers, pandemic, self report, personal protective equipment

## Abstract

The dental profession has endured unprecedented disruption amid COVID-19. Novel stressors have included a high risk of occupational exposure to COVID-19, financial losses, and stricter infection prevention and control requirements. The present study investigated the longitudinal impact of COVID-19 on the stress and anxiety levels of a cohort of Canadian dentists (*N* = 222) between September 2020 and October 2021. Salivary cortisol was selected as a biomarker of mental stress, and 10 sets of monthly saliva samples (2,131 in total) were self-collected, sent to our laboratory in prepaid courier envelopes, and analyzed by enzyme-linked immunosorbent assay. To assess COVID-19 anxiety, 9 monthly online questionnaires were administered, comprising a general COVID-19 anxiety instrument and 3 items regarding the impact of dentistry-related factors. Bayesian log-normal mixed effect models were fitted to estimate the longitudinal trajectory of salivary cortisol levels and their association with the disease burden of COVID-19 in Canada. After accounting for age, sex, vaccination status, and the diurnal rhythm of cortisol secretion, a modest positive association was found between dentists’ salivary cortisol levels and the count of COVID-19 cases in Canada (96% posterior probability). Similarly, the self-reported impact of dentistry-related factors, such as fear of getting COVID-19 from a patient or coworker, was greatest during peaks of COVID-19 waves in Canada; however, general COVID-19 anxiety decreased consistently throughout the study period. Interestingly, at all collection points, the majority of participants were not concerned about personal protective equipment. Overall, participants reported relatively low rates of psychological distress symptoms in relation to COVID-19, a result that should be reassuring for the dental community. Our findings strongly suggest a link between self-reported and biochemical measurements of stress and anxiety in Canadian dentists during the COVID-19 pandemic.

## Introduction

The COVID-19 pandemic has aroused widespread psychological distress in the Canadian population (Généreux et al. 2021), imposing a particularly severe burden on dentists. The disease’s etiologic agent, the SARS-CoV-2 virus, has been identified in saliva (To et al. 2020) and can be transmitted asymptomatically via respiratory droplets, aerosols, and splatter of oral fluid (Peng et al. 2020). The dental setting therefore presents a potentially high risk of COVID-19 cross-infection (Singhal et al. 2021). Indeed, dentists’ close interactions with patients and performance of aerosol-generating procedures have created unique challenges during this pandemic. In mid-March 2020, dental regulatory authorities across Canada ordered a cessation of routine care. By early May, clinics were permitted to reopen but with stricter infection prevention and control (IPAC) requirements (College of Dental Surgeons of Saskatchewan 2020; Ordre des dentistes du Québec 2020; Royal College of Dental Surgeons of Ontario 2020). However, literature concerning the efficacy of specific IPAC protocols in preventing COVID-19 transmission was limited, and professional guidance on the topic was regarded as inconsistent (Noushi et al. 2021). As well, the early months of the pandemic were complicated by personal protective equipment (PPE) shortages and heightened anxieties among patients and dental staff (Gohil et al. 2021; Owen et al. 2022).

Despite the rapid vaccine rollout beginning in December 2020 and the progressive lifting of public health restrictions thereafter, authorities continued urging dentists to remain vigilant (Canadian Dental Association 2021). More recent challenges have included the emergence of novel SARS-CoV-2 variants and the ongoing evolution of public health policies. Accordingly, numerous studies conducted during the pandemic have identified high levels of self-reported mental health symptoms among dentists (Alencar et al. 2021; Cimilluca et al. 2021; Gohil et al. 2021; Ismail et al. 2021; Özarslan and Caliskan 2021; Ranka and Ranka 2021; Owen et al. 2022; Salehiniya et al. 2022). However, these studies were missing an objective biological indicator, such as salivary cortisol, to quantify psychological distress. As well, their cross-sectional designs prevented the assessment of temporal changes in mental health symptoms as the pandemic evolved.

Prepandemic literature had already reported high rates of suicidality, burnout, stress, anxiety, and depression among dentists (Rada and Johnson-Leong 2004; Collin et al. 2019). With the addition of pandemic-related stressors, there has been a pressing need for further mental health research in the profession. The present study aims to use monthly self-reports and salivary cortisol measurements to evaluate the psychological impact of COVID-19 on a sample of Canadian dentists over 1 y. Our specific objectives are to estimate 1) the average trajectory of salivary cortisol levels among Canadian dentists from September 2020 to October 2021; 2) the extent to which COVID-19 disease burden is associated with salivary cortisol levels in the study population during this period; and 3) the correlation between salivary cortisol levels and self-reported COVID-19 anxiety.

## Materials and Methods

### Study Design

We used data from a prospective cohort study designed to estimate the incidence of COVID-19 among Canadian dentists. Detailed methods of the study have been published elsewhere (Madathil et al. 2022). Briefly, a cohort of practicing dentists across 9 provinces of Canada was established in late July 2020 and followed for 1 y. Informed consent and demographic information were obtained with an online form, and monthly questionnaires were completed during 9 collection points from November 2020 to October 2021. A subset of participants also provided monthly self-collected saliva samples during 10 collection points from September 2020 to October 2021. Only those who provided questionnaire responses and saliva samples (*N* = 222) were analyzed in the present study. Ethics approval was granted by the University of Saskatchewan and McGill University Research Ethics Boards (BIO ID 2553 and A06-M49-20A, respectively).

### Saliva Collection and Analysis

Cortisol is a steroid hormone that is measurable in all bodily fluids. Its secretion follows a diurnal rhythm, peaking in the early morning and declining thereafter (Vining et al. 1983). Acute mental stress stimulates a rise in cortisol levels that is independent of biological rhythms; as such, cortisol has proven to be a reliable biomarker of mental stress (Smyth et al. 2013). Saliva is the optimal biofluid for cortisol quantification due to the ease of self-collection and its correlation with the biologically active free fraction of cortisol in serum (Vining et al. 1983). Moreover, saliva self-collection is relatively stress-free, circumventing the artificial increase in cortisol levels that may occur during phlebotomy.

Whole saliva was self-collected with a swab-based collection kit (Super•SAL; Oasis Diagnostics) and squeezed into a tube containing RNA stabilizer (RNAlater; Sigma-Aldrich). Samples were immediately sealed, labeled, and transported in prepaid courier envelopes to the Salivary Proteomics Research Laboratory, University of Saskatchewan. Upon receipt, samples were promptly stored in a secure −80 °C freezer located within the laboratory. As part of the original study (Madathil et al. 2022), each sample was thawed and tested for the presence of SARS-CoV-2.

Before performing cortisol analyses, we conducted a quality control experiment to confirm that the presence of RNA stabilizer did not interfere with cortisol quantification. Saliva samples were then centrifuged at 1,500 × *g* for 15 min at 4 °C. Aliquots of supernatant were transferred into new microcentrifuge tubes, allowed to reach room temperature, and vortexed immediately prior to analysis. Salivary cortisol (μg/dL) was quantified in duplicate via a competitive enzyme-linked immunosorbent assay kit according to manufacturer instructions (Salimetrics 2021). Optical densities were recorded with a spectrophotometer at 450 nm. Positive and negative controls were included with each microtiter plate, and all samples were analyzed within 6 mo of receipt.

### Self-report Data Collection

An online baseline survey was administered via the LimeSurvey platform to collect relevant demographic and dental practice information. Participants then completed monthly 12-item questionnaires (Appendix Table 1) wherein they rated their anxiety levels during the 2 wk prior to their last working day. The psychometrically validated COVID-19 Anxiety Syndrome Scale (C-19ASS; Nikčević and Spada 2020) was used to quantify general COVID-19 anxiety or anxiety not specific to dentists’ professional lives. This 2-factor instrument focuses on perseverate thinking and avoidance behaviors. The remaining 3 items were developed to evaluate the impact of dentistry-related factors amid COVID-19. With the same Likert scale as the C-19ASS, these items were tested for face validity among dentists practicing during the pandemic. Validation analyses are currently ongoing, testing the hypothesis that these new items can be incorporated into a modified C-19ASS for dentists. To compare the biochemical and self-reported measures of stress and anxiety, we analyzed the subset of participants who provided a saliva sample during the 2 wk prior to their questionnaire completion date.

### Statistical Analysis

The descriptive statistical analysis comprised means and standard deviations for continuous variables and sample proportions for categorical variables. To estimate the longitudinal change in salivary cortisol levels during the study period, we fitted a bayesian log-normal mixed effect model. The mixed effect model allowed us to consider the correlation due to repeated measures within a participant by including a random intercept. We used a thin plate regression spline to model the time trend, thus allowing for nonlinear trajectories.

We extracted data on the count of COVID-19 cases reported in Canada (7-d moving average) from https://health-infobase.canada.ca/covid-19/. A bayesian log-normal mixed effect model with random intercept was used to estimate the association between total COVID-19 case counts in the 7 d prior to saliva collection and salivary cortisol levels. Given the lack of relevant literature, it was unclear whether changes in cortisol levels would be anticipatory, in phase, or lagging with respect to COVID-19 cases. With the rapidly evolving nature of the pandemic, however, time deviations were expected to be small, and no phase adjustments were performed.

In both models, to account for the diurnal rhythm of cortisol secretion, the time of sample collection was included as a thin plate spline. All models were adjusted for age, sex, and vaccination status. The estimated average longitudinal trajectory of salivary cortisol levels and the diurnal rhythm of salivary cortisol levels are reported with their corresponding 95% credible intervals. Weekly informative priors were used for all parameters in the model. We used RStan (Stan Development Team 2022) and brms (Bürkner 2017) packages in R to fit the models. Details of the models, including the code, are provided at Github (https://github.com/MadathilSA/salivary_cortisol_among_dentists).

In the subset of participants who provided a saliva sample within 2 wk of their questionnaire completion date, we compared C-19ASS perseveration and avoidance scores with salivary cortisol levels using Spearman rank order correlation.

## Results

Demographic and dental practice characteristics of the study cohort are presented in the Table. Apart from having a higher proportion of female participants, these characteristics are comparable to national data obtained from the Canadian Dental Association on July 30, 2020 (Appendix Table 2). During the study period, 218 of 222 participants (98%) received at least 1 dose of a COVID-19 vaccine.

**Table.** Demographic and Dental Practice Characteristics of the Study Cohort (*N* = 222).

Characteristic	*n*	%
Sex		
Female	125	56.3
Male	97	43.7
Age, y		
20 to 30	15	6.8
>30 to 40	45	20.3
>40 to 50	67	30.2
>50 to 60	70	31.5
>60 to 70	23	10.4
>70 to 80	2	0.9
Vaccination status		
Vaccinated	218	98.2
Nonvaccinated	4	1.8
Dental license type		
General practitioner	207	93.2
Specialist	15	6.8
Location of primary dental practice		
Ontario	77	34.7
Québec	55	24.8
British Columbia	45	20.3
Alberta	14	6.3
Nova Scotia	9	4.1
Saskatchewan	8	3.6
Manitoba	8	3.6
Prince Edward Island	5	2.3
Newfoundland and Labrador	1	0.5
Community served by primary dental practice		
Metropolitan	43	19.4
Urban	77	34.7
Suburban	62	27.9
Rural	39	17.6
Remote	1	0.5

**Figure 1.**
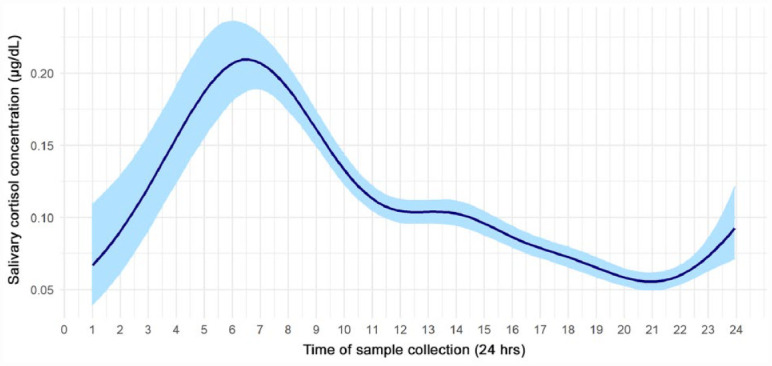
Diurnal rhythm of salivary cortisol (μg/dL) in all samples acquired during the study period, estimated via a bayesian log-normal mixed effect model. Light blue shading represents the 95% credible interval. Our data demonstrate a typical diurnal rhythm with a peak around 7 am and a steady decrease over the course of the day.

The majority of participants (82%) provided all 10 saliva samples and completed all 9 questionnaires (95%). A total of 2,165 saliva samples were collected, 34 of which contained insufficient volume; thus, 2,131 samples were analyzed. Twenty-six percent of saliva samples were collected on the same day as questionnaire completion (Appendix Figure). All samples were COVID-19-negative, and only 1 participant reported contracting COVID-19 during the study period (6 d prior to saliva collection). The coefficients of variability between replicate cortisol measurements were consistent with ideal values provided by the assay kit manufacturer (Salimetrics 2021). The diurnal rhythm estimated from the model after adjusting for age, sex, follow-up, and vaccination status is presented in Figure 1. In agreement with existing literature (Vining et al. 1983), cortisol levels peaked around 7 am and declined throughout the day.

**Figure 2.**
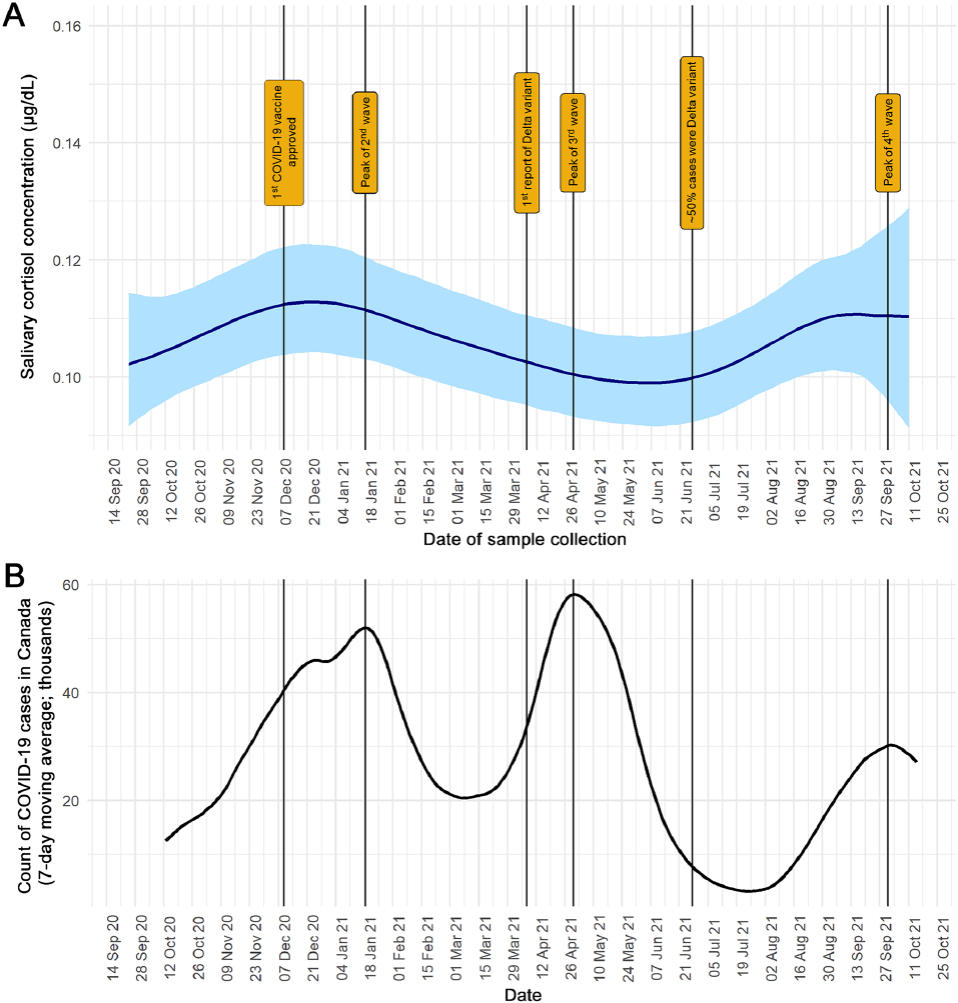
A modest positive association was observed between the average trajectory of dentists’ salivary cortisol levels (μg/dL) and the count of COVID-19 cases in Canada (96% posterior probability). Vertical lines represent important milestones during the COVID-19 pandemic in Canada. (**A**) A bayesian log-normal mixed effect model was used to estimate the average trajectory of salivary cortisol levels throughout the study period. Light blue shading represents the 95% credible interval. (**B**) Count of COVID-19 cases in Canada (7-d moving average) during the study period, re-created with data from https://healthinfobase.canada.ca/covid-19/. Note that salivary cortisol levels peaked in January and September 2021, in accordance with the peaks of the second and fourth waves of COVID-19 in Canada. However, there was a high degree of variability in salivary cortisol levels.

The estimated average longitudinal trajectory of salivary cortisol levels is presented in Figure 2. The general trend in cortisol levels corresponded with changes in the disease burden of COVID-19 in Canada, in that higher cortisol levels were found during peaks of COVID-19 waves. Moreover, based on the bayesian log-normal mixed effect model, there was a 96% posterior probability of a positive association between the average trajectory of salivary cortisol levels and the total count of COVID-19 cases in Canada during the 7 d prior to saliva collection. An increase of 10,000 total COVID-19 cases was associated with an increase of 0.01 μg/dL of salivary cortisol (95% credible intervals = −0.002 to 0.02).

**Figure 3.**
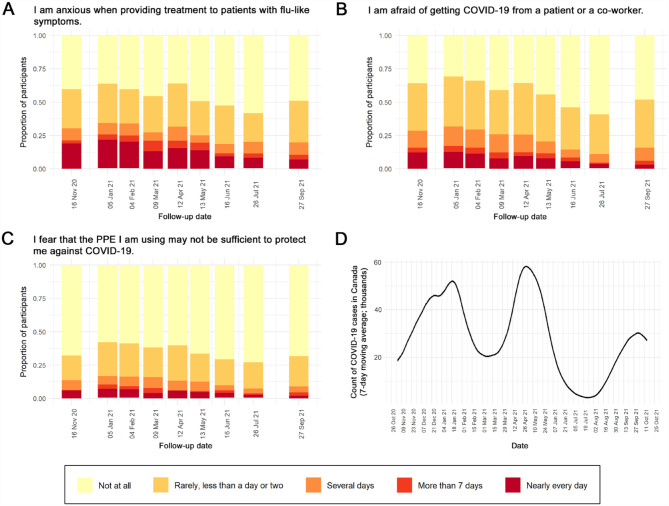
(**A**–**C**) Self-reported impact of dentistry-related factors among a cohort of Canadian dentists. Participants were asked to rate their experiences during the past 2 wk on a 5-point time-anchored scale (0 = *not at all*, 4 = *nearly every day*). Each vertical column represents the responses provided during a single collection point. (**D**) Count of COVID-19 cases in Canada (7-d moving average) during the study period, re-created with data from https://health-infobase.canada.ca/covid-19/. For all 3 dentistry-related factors, the proportion of participants reporting at least some degree of impact peaked in January, April, and September 2021. The timing of these peaks corresponds with that of the second, third, and fourth waves of COVID-19 in Canada.

The self-reported impact of all dentistry-related factors examined in this study displayed similar distributions (Fig. 3A–C)—that is, the proportion of participants reporting at least some degree of impact peaked in January, April, and September 2021. This pattern is highly congruent with the disease burden of COVID-19 in Canada, in that the second, third, and fourth waves of the pandemic (Fig. 3D) peaked during the same months. However, the overall impact of dentistry-related factors can be considered low throughout the study period, as the most frequent responses to all items at all time points were *not at all* and *rarely, less than a day or two*.

During the first 6 questionnaire collection points, the majority of participants reported anxiety about getting COVID-19 from a patient or coworker and fear about treating patients with flu-like symptoms (Fig. 3A, B). Throughout the study period, however, most participants were confident that their PPE was sufficient to protect them against COVID-19 (Fig. 3C).

**Figure 4.**
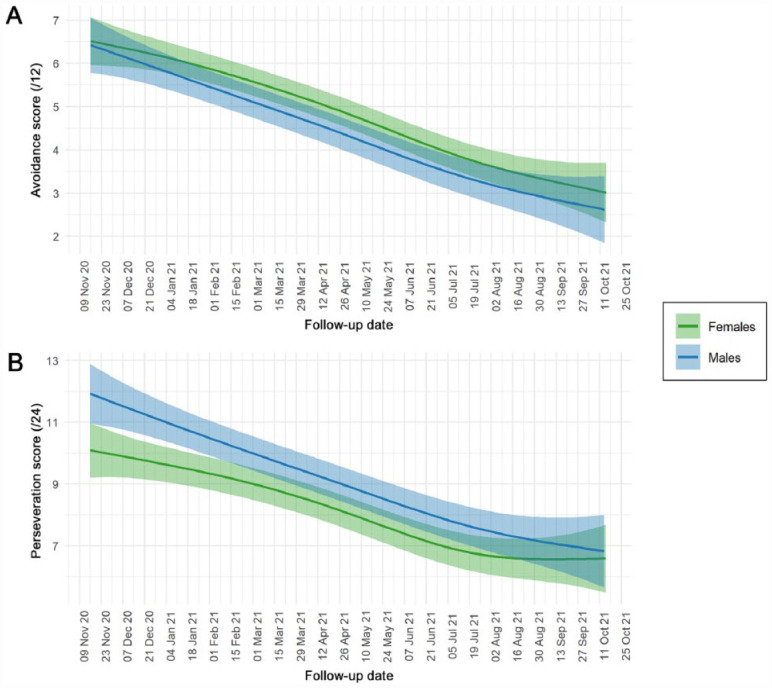
Average trajectory of general COVID-19 anxiety scores stratified by sex. Participants were asked to consider their experiences during the past 2 wk and rate COVID-19 Anxiety Syndrome Scale items on a 5-point time-anchored scale (0 = *not at all*, 4 = *nearly every day*). Green and blue shading represents the 95% credible interval for female and male participants, respectively. (**A**) Avoidance factor (range of possible scores, 0 to 12). (**B**) Perseveration factor (range of possible scores, 0 to 24). Note that both perseveration and avoidance scores consistently decreased throughout the study period.

The average trajectory of C-19ASS perseveration and avoidance scores showed a progressive decrease from November 2020 to October 2021 (Fig. 4). Only 50% of the saliva samples (*n* = 1,094) were collected within 2 wk prior to a questionnaire response, of which 26% (*n* = 285) were collected on the same day. Jointly, this amounts to 217 participants having matched data on salivary cortisol levels and questionnaire responses. Among these samples, the perseveration and avoidance scores were poorly correlated with the matched salivary cortisol levels (Spearman correlation coefficients, 0.022 and −0.029, respectively).

## Discussion

The dental profession has endured unprecedented disruption amid COVID-19. Unsurprisingly, reports throughout the pandemic have emphasized high rates of mental health symptoms in dentists (Alencar et al. 2021; Cimilluca et al. 2021; Gohil et al. 2021; Ismail et al. 2021; Özarslan and Caliskan 2021; Ranka and Ranka 2021; Owen et al. 2022; Salehiniya et al. 2022). Little information is available, however, regarding temporal changes in these symptoms. Thus, the present study aimed to characterize the longitudinal impact of COVID-19 on Canadian dentists’ stress and anxiety levels during 1 y of the pandemic (September 2020 to October 2021) using biochemical stress and self-reported anxiety measures.

This study implemented saliva self-collection and courier transportation on a large scale, having analyzed >2,000 samples from across Canada. Our approach moved from the proof-of-principle stage to practical application in the use of saliva for biomarker quantification. Our quality control data verified that sample quality was conserved during transportation, establishing this as a simple cost-effective solution for large-scale saliva research. Furthermore, the scientific and medical communities are only beginning to uncover the clinical utility of saliva (Boroumand et al. 2021). Its vast assortment of potential analytes and noninvasive collection methods make saliva an appealing biofluid for examining oral and systemic conditions. As such, we expect saliva self-collection and courier transportation to be used in the future for myriad diagnostic and prognostic applications.

After accounting for age, sex, vaccination status, and the diurnal rhythm of cortisol secretion, our results indicate a modest positive association between dentists’ salivary cortisol levels and the count of COVID-19 cases in Canada. That is, dentists experienced a greater degree of mental stress when the state of the pandemic was more severe. A similar pattern was found between the impact of dentistry-related factors (e.g., fear of getting COVID-19 from a patient or coworker) and the state of the pandemic. Together, these findings imply a link between biochemical and self-reported measurements of psychological distress in Canadian dentists. Relatedly, a study of Argentinian hospital workers identified associations among hair cortisol, stress, and pandemic-related burnout (Ibar et al. 2021). Thus, future psychological studies could benefit from the addition of an objective biological indicator such as salivary cortisol to their survey-based designs.

A comparable distribution of mental health symptoms has been identified within other health care professions. In a 12-mo, 4-wave longitudinal study, Ontario hospital workers reported a greater degree of emotional exhaustion during peaks of the COVID-19 case rate (Maunder et al. 2022). Moreover, a 4-wave study in Japan found that levels of psychological distress among health care workers varied in accordance with COVID-19 cases, whereas the general public exhibited a different pattern (Sasaki et al. 2021). Like nurses and physicians, dentists bear the responsibility of caring for patients throughout this challenging time. Shared stressors such as the risk of contracting COVID-19 and uncertainties about the future underscore the difficulty of providing health care during a pandemic.

Outside of their professional lives, however, dentists appear to be adapting to the pandemic. Indeed, general COVID-19 anxiety decreased steadily throughout the study period. This decrease was observed regardless of participants’ age, sex, or vaccination status, even though 98% of participants received a COVID-19 vaccine during this study. Similar improvements in mental health factors have been observed within the general public of various countries (Aknin et al. 2022; Benatov et al. 2022). Some authors have postulated that the general public is adapting to a “new normal” and growing desensitized to COVID-19 (Corpuz 2021; Stevens et al. 2021); based on our results, the same may be true of dentists. Overall, it is likely that dentists have drawn a distinction in how they interpret the pandemic in their high-risk professional lives versus their lives outside of dentistry.

Somewhat surprising, the majority of participants reported no concerns about PPE throughout the study period. Although fear about the efficacy of PPE varied in accordance with COVID-19 case counts, this finding differs from reports earlier in the pandemic. For example, a survey of Polish dentists in April 2020 found that 84% of participants who discontinued dental care were concerned about insufficient PPE (Tysiąc-Miśta and Dziedzic 2020). Similarly, 69.5% of participants in a cohort of Pakistani dentists reported PPE as a source of stress and anxiety (Kamal et al. 2021). We speculate that the publication of relevant literature and updating of professional guidance have improved dentists’ confidence in their office IPAC protocols. As well, the incidence of COVID-19 among Canadian dentists was recently found to be lower than that of the general public, an achievement largely attributed to PPE and other IPAC measures (Madathil et al. 2022).

Despite significant variability, the improvements over time in general COVID-19 anxiety and the relatively low impact of all dentistry-related factors in this study should be reassuring for the dental community. This is in contrast to all known studies conducted earlier in the pandemic, which reported worrisome levels of COVID-19–related psychological distress among dentists. In early 2020, the prevalence of anxiety disorders among dentists in China was 4.3 times that of the general public (Zhao et al. 2020). In mid-2020, 47.3% of dentists in Brazil reported experiencing depression (Alencar et al. 2021). In early 2021, 43% of Welsh dentists expressed an inability to cope with the daily stress of their job (Owen et al. 2022). The importance of promoting mental health in dentistry cannot be understated. It is well established that psychological distress among health care providers is associated with reduced job satisfaction, lower patient satisfaction, and poorer clinical outcomes (Leiter et al. 1998; Le et al. 2021; Yansane et al. 2021). Therefore, as the profession transitions from the early stages of COVID-19 toward a new normal, continued efforts must be taken to facilitate mental well-being in dentistry.

Due to a limited sample size, this study was unable to compare between general practitioners and specialist dentists or among dentists in different provinces. Future research would also benefit from the addition of a control group to permit comparisons with the general public. Moreover, the lifting of public health policies relevant to C-19ASS items limited a consistent analysis. Limitations of salivary cortisol, a well-established biomarker of mental stress, include the inability to draw between-subject comparisons, the possible confounding effects of acute stressors or medical conditions affecting the hypothalamic-pituitary-adrenal axis, and the additional time and resources necessary to conduct biochemical assays. Finally, this study may have enabled self-selection bias: email invitations were sent to the majority of our population of interest, but as with many studies conducted during the pandemic, our response rate was low. Nevertheless, the demographic and dental practice characteristics of our sample were consistent with the overall population of Canadian dentists. Furthermore, to our knowledge, there are no other prospective cohort studies that collected biochemical and self-reported measurements of psychological distress among Canadian dentists.

In summary, this study characterized the longitudinal trajectory of factors related to dentists’ mental health during 1 y of the COVID-19 pandemic. We found that dentists have remained mindful about COVID-19 in their high-risk professional lives but have been adapting to it outside of dentistry. Our findings imply a link between biochemical and self-reported measurements of stress and anxiety in Canadian dentists during the COVID-19 pandemic. In conducting this study, we demonstrated a simple and effective approach for the large-scale implementation of saliva self-collection and courier transportation.

## Author Contributions

R.J. Kolbe, contributed to data acquisition, analysis, and interpretation of the data, drafted the manuscript; S.A. Madathil, contributed to conception and design, data analysis and interpretation of the data, drafting and critically revised the manuscript; L.M. Marin, contributed to conception and design, data acquisition, analysis, and interpretation of the data, contributed to the final version of the manuscript; R. Seth, N. Faraj, P.J. Allison, C. Quiñonez, M. Glogauer, contributed to data interpretation of the data, contributed to the final version of the manuscript; W.L. Siqueira, contributed to conception and design, data expertise in salivary biochemistry and to interpretation of the data, critically revised the manuscript; M.F. Siqueira, contributed to conception and design, data and directly supervised R.J.K., critically revised the manuscript. All authors gave their final approval and agree to be accountable for all aspects of the work.

## Supplemental Material

sj-docx-1-jdr-10.1177_00220345231178726 – Supplemental material for Salivary Cortisol and Anxiety in Canadian Dentists over 1 Year of COVID-19 Click here for additional data file. Supplemental material, sj-docx-1-jdr-10.1177_00220345231178726 for Salivary Cortisol and Anxiety in Canadian Dentists over 1 Year of COVID-19 by R.J. Kolbe, S.A. Madathil, L.M. Marin, R. Seth, N. Faraj, P.J. Allison, C. Quiñonez, M. Glogauer, W.L. Siqueira and M.F. Siqueira in Journal of Dental Research
