# Supplementary material for: Salivary Cortisol and Anxiety in Canadian Dentists over 1 Year of COVID-19
Source: J Dent Res. 2023 Jun 15;102(10):1114–21. doi: 10.1177/00220345231178726 (PMC10273046; doi:10.1177/00220345231178726)
Supplement: sj-docx-1-jdr-10.1177_00220345231178726 – Supplemental material for Salivary Cortisol and Anxiety in Canadian Dentists over 1 Year of COVID-19 [file sj-docx-1-jdr-10.1177_00220345231178726.docx]

**Appendix Table 1.** COVID-19 anxiety questionnaire.

| **Instrument** | **Subscale** | **Item** |
| --- | --- | --- |
| COVID-19 anxiety syndrome scale (C-19ASS) | Avoidance | I have avoided using public transport because of the fear of contracting coronavirus (COVID-19). |
|  | Perseveration | I have checked myself for symptoms of coronavirus (COVID-19). |
|  | Avoidance | I have avoided going out to public places (shops, parks) because of the fear of contracting coronavirus (COVID-19). |
|  | Perseveration | I have been concerned about not having adhered strictly to social distancing guidelines for coronavirus (COVID-19). |
|  | Avoidance | I have avoided touching things in public spaces because of the fear of contracting coronavirus (COVID-19). |
|  | Perseveration | I have read about news relating to coronavirus (COVID-19) at the cost of engaging in work. |
|  | Perseveration | I have checked my family members and loved ones for the signs of coronavirus (COVID-19). |
|  | Perseveration | I have been paying close attention to others displaying possible symptoms of coronavirus (COVID-19). |
|  | Perseveration | I have imagined what could happen to my family members if they contracted coronavirus (COVID-19). |
| Dentistry-related factors |  | I am afraid of getting COVID-19 from a patient or a co-worker. |
|  |  | I am anxious when providing treatment to patients with flu-like symptoms. |
|  |  | I fear that the PPE I am using may not be sufficient to protect me against COVID-19. |

**Appendix Table 2.** Comparison of participant characteristics with national data obtained from the Canadian Dental Association on July 30, 2020.

| **Characteristic** | **Level** | **Our study (%)** | **Canadian Dental Association (%)** | |
| --- | --- | --- | --- | --- |
| Sex | Female | 56.3 | 40.2 |  |
|  | Male | 43.7 | 59.8 |  |
| Age | (20,30] | 6.8 | 5.1 |  |
|  | (30,60] | 82.0 | 74.2 |  |
|  | (60,80] | 11.3 | 20.7 |  |
| Dental licence type | General practitioner | 93.2 | 87.9 |  |
|  | Specialist | 6.8 | 12.1 |  |
| Province of primary dental practice | Ontario | 34.7 | 43 |  |
|  | Québec | 24.8 | 21.1 |  |
|  | British Columbia | 20.3 | 14.7 |  |
|  | Alberta | 6.3 | 11 |  |
|  | Nova Scotia | 4.1 | 2.3 |  |
|  | Saskatchewan | 3.6 | 2.1 |  |
|  | Manitoba | 3.6 | 3 |  |
|  | Prince Edward Island | 2.3 | 0.3 |  |
|  | Newfoundland and Labrador | 0.5 | 0.9 |  |
|  | New Brunswick | 0.0 | 0.1 |  |
|  | Northwest Territories | 0.0 | 0.1 |  |
|  | Nunavut | 0.0 | 1.3 |  |
|  | Yukon | 0.0 | 0.1 |  |


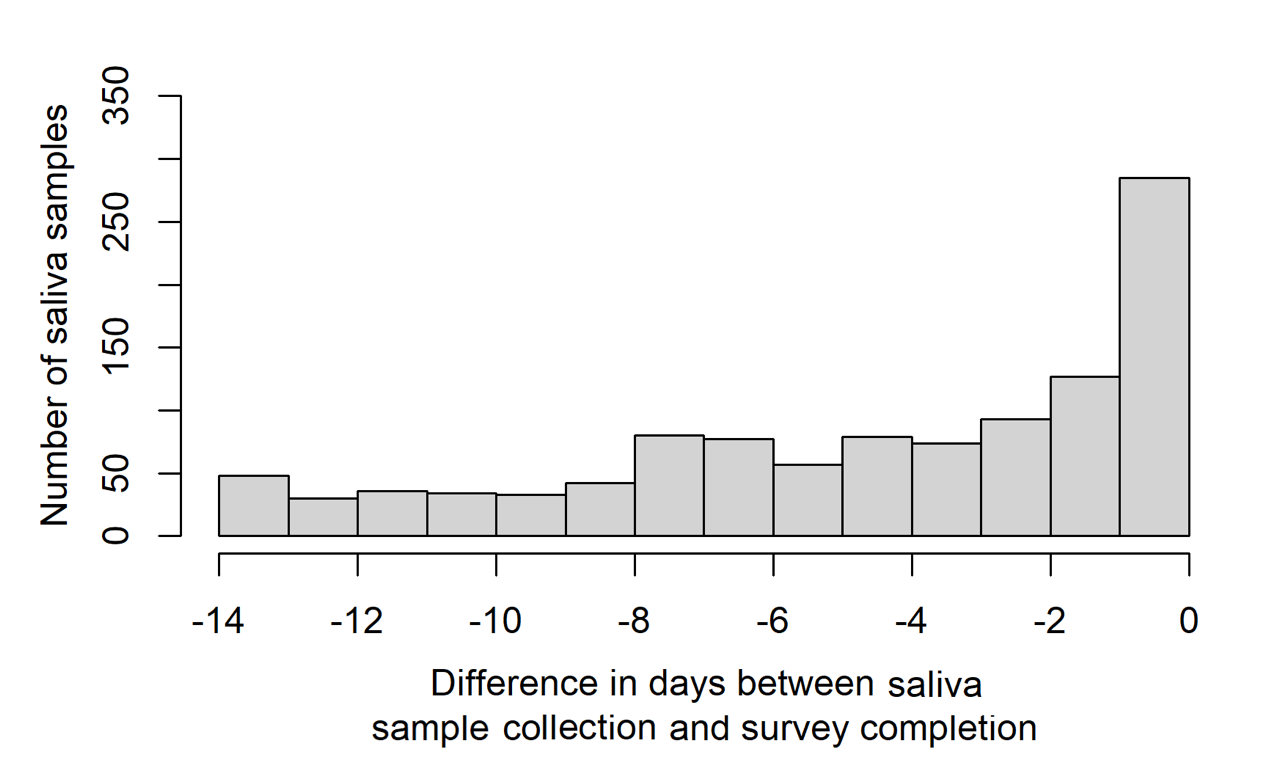


**Appendix Figure 1.** Distribution of the differences in time between saliva sample collection and survey completion (days).
